# Robotic Cochlear Implant Surgery: Imaging-Based Evaluation of Feasibility in Clinical Routine

**DOI:** 10.3389/fsurg.2021.742219

**Published:** 2021-09-29

**Authors:** Alice Barbara Auinger, Valerie Dahm, Rudolfs Liepins, Dominik Riss, Wolf-Dieter Baumgartner, Christoph Arnoldner

**Affiliations:** Department of Otorhinolaryngology, Head and Neck Surgery, Medical University of Vienna, Vienna, Austria

**Keywords:** cochlear implantation, robotic surgery, robotic cochlear implantation, minimal invasive surgery, keyhole access

## Abstract

**Background:** Robotic surgery has been proposed in various surgical fields to reduce recovery time, scarring, and to improve patients' outcomes. Such innovations are ever-growing and have now reached the field of cochlear implantation. To implement robotic ear surgery in routine, it is of interest if preoperative planning of a safe trajectory to the middle ear is possible with clinically available image data.

**Methods:** We evaluated the feasibility of robotic cochlear implant surgery in 50 patients (100 ears) scheduled for routine cochlear implant procedures based on clinically available imaging. The primary objective was to assess if available high-resolution computed tomography or cone beam tomography imaging is sufficient for planning a trajectory by an otological software. Secondary objectives were to assess the feasibility of cochlear implant surgery with a drill bit diameter of 1.8 mm, which is the currently used as a standard drill bit. Furthermore, it was evaluated if feasibility of robotic surgery could be increased when using smaller drill bit sizes. Cochlear and trajectory parameters of successfully planned ears were collected. Measurements were carried out by two observers and the interrater reliability was assessed using Cohen's Kappa.

**Results:** Under the prerequisite of the available image data being sufficient for the planning of the procedure, up to two thirds of ears were eligible for robotic cochlear implant surgery with the standard drill bit size of 1.8 mm. The main reason for inability to plan the keyhole access was insufficient image resolution causing anatomical landmarks not being accurately identified. Although currently not applicable in robotic cochlear implantation, narrower drill bit sizes ranging from 1.0 to 1.7 mm in diameter could increase feasibility up to 100%. The interrater agreement between the two observers was good for this data set.

**Discussion:** For robotic cochlear implant surgery, imaging with sufficient resolution is essential for preoperative assessment. A slice thickness of <0.3 mm is necessary for trajectory planning. This can be achieved by using digital volume tomography while radiation exposure can be kept to a minimum. Furthermore, surgeons who use the software tool, should be trained on a regular basis in order to achieve planning consistency.

## Introduction

According to the World Health Organization, 430 million people require hearing rehabilitation due to hearing loss (WHO, 2021).^1^ For people with no functional hearing, cochlear implantation (CI) has become the standard treatment for hearing rehabilitation (1). The standard procedure is a cortical mastoidectomy followed by a posterior tympanotomy. Both steps require extensive drilling of the mastoid bone. Additionally, during posterior tympanotomy, the facial nerve is at risk of injury. Within the last 30 years, the use of robotics for minimal invasive surgeries has been growing in various surgical fields such as orthopedic hip replacements, laparoscopic cholecystectomies, or urological, cardiological and transoral procedures (2–5). Recently, minimal invasive surgical techniques have been proposed for middle and inner ear access in order to reduce the extent of the surgical approach such as a direct access to the round window region originating from the surface of the mastoid and without performing a mastoidectomy (6, 7). Several studies using cadaveric specimens have proven feasibility of a robot to perform neurotological surgeries (8–11).

For robotic CI surgery, high expectations are raised for preserving residual hearing. The patients' outcome and hearing performance might be improved due to a reduced trauma to the inner ear. By eliminating the surgeon's tremor, more consistent insertion techniques can be achieved with a robot compared to manual insertion (12, 13). Labadie et al. reported on a stereotactic frame-based robotic CI surgery (14). Based on the robot developed by Bell et al. (11), CI surgery was later performed in a patient with a task-specific robotic system including computer-assisted surgery planning, intraoperative stereotactic image guidance, and multipolar facial neuromonitoring (15, 16). Since then, a few adult patients have been successfully implanted with this technique in Europe (17). CE mark for the so-called HEARO robot (CAScination AG, Bern, Switzerland and MED-EL GmbH, Innsbruck, Austria) was obtained in March 2020 for the use in patients above the age of 18 years (17). Using the HEARO system, a tunnel bordered by the facial nerve and chorda tympani is directly drilled through the mastoid to the round window (11, 15, 17). While the facial nerve is often skeletonized in conventional CI surgery, there is no direct visualization during robotic CI surgery. For the HEARO procedure, several safety steps are currently implemented (15, 17) and with current facial nerve monitoring using multipolar stimulation probes, sufficient safety distance margins ≥0.4 mm can be correctly identified (18). Safety margins < 0.4 mm can be achieved without structural nerve damage, but whether the nerve's functional integrity can be preserved, remains unclear in clinical application (19).

The first step in robotic CI surgery is to assess a safe path for the drill through the facial recess. A surgical planning software is used to segment the middle and inner ear anatomy with manual, semiautomated and fully automated tools (20). Semiautomated instruments calculate anatomic models based on selected points on image data by the examiner, which can be completed within a few minutes. It can be also used to measure the cochlear duct length (CDL) and electrode visualization aids the surgeon to choose the most suitable electrode array. Consequently, complications such as incomplete insertion, tip fold-over or kinking can be reduced. In contrast, planning via manual segmentation is time consuming and has to be done by an experienced examiner.

So far, the robotic procedure has been done in only a few patients and its applicability in clinical routine needs yet to be assessed. The first step in preparing for robotic CI surgery is checking the feasibility based on the individual anatomy. Trajectory planning has to be performed on an otological software with uploaded image data (computed tomography or cone-beam tomography) in order to assess the ideal path to the round window, starting from the surface of the mastoid through the facial recess and to the middle ear. Consequently, it has to be evaluated if clinically available image data is sufficient for planning or if adaptations to the preoperative assessment are necessary. In this study, we evaluated the possibility of robotic CI surgery based on clinically available imaging. Results of the current study should improve preoperative management of CI candidates in order to fulfill all criteria for robotic CI surgery.

## Materials and Methods

### Patients

Fifty patients (100 ears) with existing preoperative computed tomography (CT) scans who were planned for CI surgery at our department were consecutively screened for the study. Based on the clinically available preoperative CT scan, a trajectory path to the round window was assessed with OTOPLAN (CAScination AG, Bern, Switzerland in collaboration with MED-EL GmbH, Innsbruck, Austria), an otological planning software used on a computer tablet or a computer desktop. Preoperative imaging was usually performed in external and different radiological institutes and therefore, quality of image data differs. As the study was primarily performed to assess feasibility of planning a trajectory path to the round window, we did not exclude any patients in advance. All consecutive patients planned for CI surgery were included, whether they were adults, children, had chronic middle ear disease, were previously implanted with any type of hearing prosthesis or showed malformations. The study was approved by the local institutional review board (1620/2019) and the study was conducted according to the ethical standards of the Helsinki Declaration (21).

### Procedures

Imaging files of preoperative CT scans were transferred to OTOPLAN in Digital Imaging and Communications in Medicine (DICOM) file format. For robotic CI surgery, a trajectory tunnel to the round window can be preoperatively planned with the software. Furthermore, the CDL can be assessed to enable individualized CI surgery in terms of choosing the correct electrode array length. Therefore, OTOPLAN guides segmentation of anatomic landmarks and enables 3D reconstruction of middle and inner ear structures based on selected points on CT images by an examiner. The software is fully compatible with the HEARO cochlear implant surgical robot. Postoperatively, the software allows for an anatomy-based fitting if the actual location of each electrode within the cochlear is displayed on cone beam CT.

Two examiners (observer 1 and observer 2) first checked if they could perform software-guided segmentation of anatomical landmarks based on image properties. In cases in which relevant anatomical structures could be sufficiently defined, planning of a 3D ear and a trajectory to the round window was performed. Both examiners were well trained in the use of the software and had experience for at least two years. Observer 1 was the first author of the study, observer 2 was an engineer of MED-EL (MED-EL GmbH, Innsbruck, Austria) who also instructs surgeons with the use of the software.

The trajectory for CI surgery was planned based on the instructions by the software. Both observers were blinded to the results of the other one and measured both ears of each patient independently. The software calculates 3D models of anatomic structures after manual selection of the following anatomical structures: the ear canal, incus, malleus, stapes, facial nerve, chorda tympani, sigmoid sinus, temporal bone and the cochlea. For assessing cochlear parameters, the examiner had to define the cochlear view (center of the modiolus, the basal turn of the cochlear, the round window in the axial, coronal and sagittal view, see Figure 1A). Cochlear parameters such as the diameter, width, height and the cochlear duct length were calculated by the software based on points selected by the examiner (round window, lateral, inferior and superior walls of the cochlear). Finally, a virtual trajectory to the round window was automatically calculated for a drill bit size of 1.8 mm which is the currently used as the standard size of the HEARO robotic system (see Figure 1B). A successful access to the cochlea was possible if sufficient safety margins of critical structures were maintained, which is a minimum of 0.4 mm to the facial nerve and 0.3 mm to the chorda tympani. Consequently, a facial recess of at least 2.5 mm is necessary to access the middle ear with a standard drill bit of 1.8 mm. If the output of an automated trajectory was not possible, adjustments were made. If there was still no safe access possible with the standard drill bit, the data was set as “not possible with standard drill bit.” The next steps included evaluation of a safe access to the middle and inner ear with narrower drill bit sizes in 0.1 mm steps ranging from 1.7 to 1.0 mm as determined by the software. However, these calculations using smaller drill bits are more of a theoretic interest for future applications, as the HEARO procedure is based on the use of 1.8 mm drill bits as of today. If a safe trajectory could be automatically computed by the software, the data was set as “possible with < 1.8 mm drill bit.” For all successfully planned trajectories, the distance to relevant anatomical structures such as the facial neve, the chorda tympani, the ossicles, and the external auditory canal was noted. All distances were automatically calculated by the software. Furthermore, the in- and out-plane angles, also automatically calculated, were assessed. The software displays the in-plane angle as offset between an ideal trajectory and the planned trajectory with respect to the plane of the basal turn with a given target (round window) (22). The out-plane angle is computed as the offset between the planned trajectory and an ideal trajectory in the plane orthogonal to the basal turn. For cases in which a sufficient software-guided planning procedure was not possible, no such parameters were collected.

**Figure 1A:**
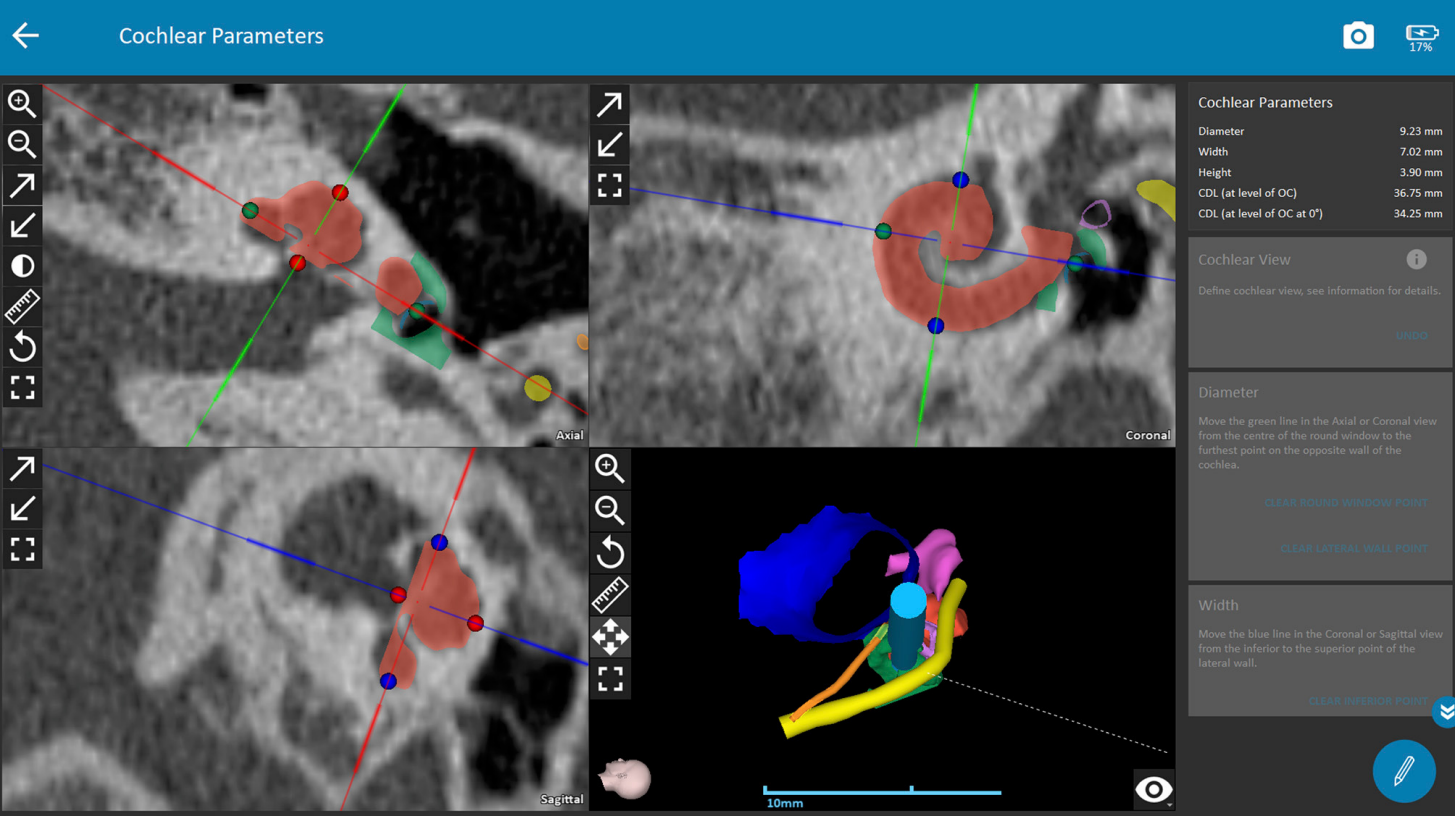
Planning procedures with OTOPLAN. Cochlear view for assessing the software-guided cochlear parameters. The observer defines the cochlear view which corresponds to the center of the modiolus, the basal turn of the cochlear, and the round window in the axial, coronal and sagittal view. The diameter, height, width and length of the cochlear are then defined by the observer based on instructions of the software. Consequently, the software automatically calculates the cochlear parameters. The right lower picture displays a 3D model of the planned trajectory. Green dots (selection of round window and lateral wall), blue dots (selection of superior and inferior wall in coronal view), red dots (inferior and superior walls of cochlear in axial view), red shading (cochlear segmentation automatically calculated by the software), green shading (bony overhang automatically calculated by the software), yellow shading (facial nerve), dark blue shading (external ear canal), pink shading (ossicles), orange shading (chorda tympani), light blue shading (drill in position of the automated trajectory).

**Figure 1B:**
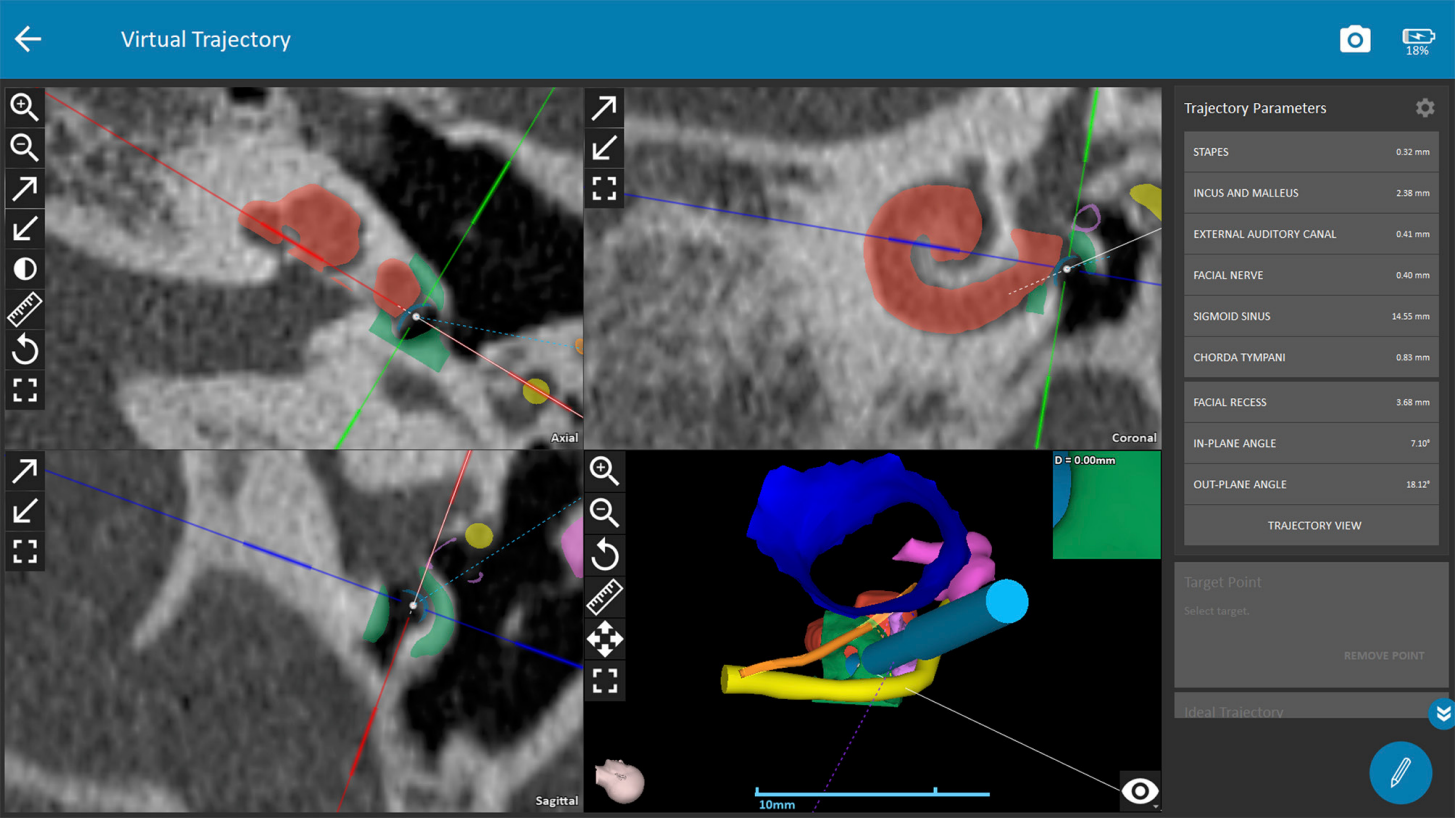
Planning procedures with OTOPLAN. The trajectory path to the middle ear/round window is calculated automatically and safety distances to critical anatomic structures are displayed by the software. Red shading (cochlear segmentation automatically calculated by the software), green shading (bony overhang automatically calculated by the software), yellow shading (facial nerve), dark blue shading (external ear canal), pink shading (ossicles), orange shading (chorda tympani), light blue shading (drill in position of the automated trajectory).

### Statistics

Data of 50 patients (100 ears) were included in the study. Each ear was planned by two observers revealing 200 measured ears. The primary goal of the study was to assess how many of the available datasets were suitable for robotic CI surgery using the standard drill bit size. Secondary objective was to expand the possibility of robotic CI surgery based on the available imaging data if narrower drilling bits were used. Descriptive statistics, i.e., mean and standard deviation (SD) were computed for ear parameters. The interrater reliability between the two observers was assessed based on Cohen's Kappa. A Cohen's Kappa (K) of ≤ 0.1 corresponds to no agreement, 0.1 < K ≤ 0.4 weak agreement, 0.4 < K ≤ 0.6 good agreement, 0.6 < K ≤ 0.8 strong agreement and 0.8 < K ≤ 1 complete agreement.^2^ Statistical analysis was performed using MATLAB (The Mathworks, Inc., Natick, USA).

## Results

**Figure 2:**
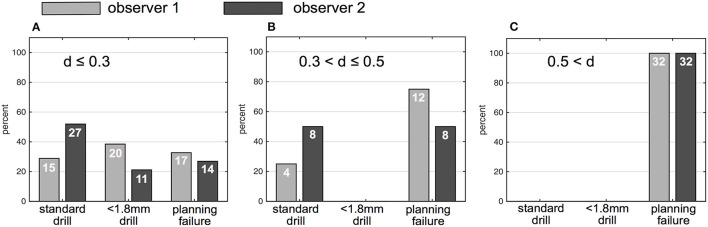
Percentage and numbers (in white) of successfully planed ears based on drill bit size (1.8 and < 1.8 mm) and percentage of unsuccessfully planned ears. **(A)** Ears planned on CT with ≤ 0.3 mm slice thickness. **(B)** Ears planned based on CT with a slice thickness between 0.3 and ≤ 0.5 mm. **(C)** Ears planned based on CT with a slice thickness > 0.5 mm; gray bars indicate results of observer 1, black bars indicate results of observer 2; d (slice thickness).

Image data of 100 ears from 50 patients (26 males, 24 females) with a mean age of 51 +/−23 years were independently analyzed by the two observers. Image resolution ranged from 0.1 x 0.1 x 0.1 mm^3^ to 0.6 x 0.6 x 1.5 mm^3^. For observer 1, 39 out of 100 ears (39%) were rated as sufficient in order to perform the software-guided planning procedure, whereas observer 2 rated data of 46 ears (46%) as sufficient. Consequently, computation of cochlear and trajectory parameters was not possible in the remaining ears and reasons for planning failures are depicted in Table 1. Image slice thickness was categorized in three groups (slice thickness ≤ 0.3 mm, > 0.3 and ≤ 0.5 mm, > 0.5 mm). Percentages of useful imaging quality according to slice thickness group are depicted in Figure 2. None of the scans with a slice thickness of > 0.5 mm enabled assessment of a safe virtual trajectory to the round window. A minimum distance to critical anatomic landmarks has to be maintained for successful planning. Mean distances to certain important structures are shown in Table 2.

**Table 1:** Reasons for trajectory planning failures.

	**Examiner 1**	**Examiner 2**
Bad image resolution	37 (60.7%)	36 (66.7%)
Software failure	9 (14.8%)	5 (9.3%)
Incomplete 3D ear	9 (14.8%)	9 (16.7%)
Corrupted image	6 (9.8%)	4 (7.4%)
	61	54

*The number and percentage (%) of planning failures based on depicted reasons. An incomplete 3D ear was existent in cases in which anatomical landmarks could not be sufficiently annotated (i.e. malformations of the middle ear or a preexisting CI on the contralateral ear in one case)*.

**Table 2:** Cochlear parameters and distance of planned trajectory to critical anatomic structures.

	**Observer 1**		**Observer 2**		
	**mean (mm)**	**SD**	**mean (mm)**	**SD**	**absolute** **difference**
**Distance of trajectory to:**					
Stapes	0.55	0.5	0.64	0.48	0.09
Incus/Malleus	2.46	0.98	2.63	0.76	0.17
External ear canal	1.33	0.92	1.44	0.61	0.11
Facial nerve	0.41	0.02	0.41	0.02	0.00
Chorda tympani	0.65	0.54	0.70	0.38	0.05
Facial recess	3.04	0.58	3.13	0.46	0.09
In-plan angle	9.21	8.43	5.37	6.38	3.84
Out-plane angle	17.48	6.76	19.41	5.15	1.93
**Cochlear parameters:**					
Cochlear diameter	9.44	0.47	9.16	0.46	0,28
Cochlear width	6.86	0.32	6.76	0.32	0.10
Cochlear height	3.79	0.30	3.88	0.25	0,09
CDL	36.48	1.57	35.16	1.56	1.32

*Means are depicted in millimeters. SD, standard deviation; CDL, cochlear duct length*.

Observer 1 could plan a safe trajectory in 19 ears (48.7%,) out of 39 sufficiently measurable images. Observer 2 successfully planned 35 out of 46 sufficiently measurable (76.1%) ears. Both these measurements were carried out using the 1.8 mm standard drill bit size. Measurements were then repeated with smaller drill bit sizes with 0.1 mm steps (1.0–1.7 mm). Consequently, the feasibility of the HEARO procedure could be increased to 100% of patients rated as sufficient for the planning procedure. Table 3 depicts successfully planned cases based on the used drill bit size as suggested by the planning software. The calculations for the interrater reliability (trajectory planning) revealed 0.52 corresponding to a good agreement.

**Table 3:** Success rate of planning a safe trajectory for the HEARO procedure.

**Drill bit size**	**Cases feasible for HEARO**	
	**Observer 1**	**Observer 2**
1.8 mm (standard size)	19 (48.7%)	35 (76.1%)
1.7 mm	+3 (56.4%)	+1 (78.3%)
1.6 mm	+4 (66.7%)	+3 (84.8%)
1.5 mm	+5 (79.5%)	+1 (87.0%)
1.4 mm	+2 (84.6%)	+2 (91.3%)
1.3 mm	+2 (89.7%)	+2 (95.6%)
1.2 mm	+2 (94.8%)	+1 (97.8%)
1.1 mm	+1 (97.4%)	+1 (100%)
1.0 mm	+1 (100%)	0
	39	46

*Successfully planned ears are depicted as number and total (percentage) for different drill bit sizes. Feasibility could be increased to 100% with including narrower drill bit sizes as small as 1.00 mm*.

The mean safety distances achieved in successfully planned trajectories (including all drill bit sizes) as well as the diameter of the facial recess, the in- and out-plane angles are depicted in Table 2. Cochlear parameters such as the cochlear diameter, height, width, and length are also reported as mean and standard deviation in Table 2.

## Discussion

With an increasing application of robotics in ear surgery, it is of interest how patients should be properly prepared for surgery. One essential step is preoperative planning of the trajectory path to the region of interest, which is the round window in case of CI surgery. The primary goal of the study was to assess feasibility of robotic CI surgery based on clinically available data of CT scans, which are mostly performed in external radiologic institutes and have therefore different image quality.

The standard drill bit size used by the HEARO robot is currently 1.80 mm in diameter. In this study and depending on the examiner, up to two thirds of measured ears were eligible for the HEARO procedure with the standard drill bit if image data was rated as sufficient. Williamson et al. created a statistical model in which approximately 46.7% of the population could accommodate necessary safety regions with a standard drill bit of 1.8 mm and a CT slice thickness of 0.2 mm (23), which is similar to our results. The smallest drill bit size the software offers is 1 mm. In the current study population, feasibility of robotic CI surgery could be increased if narrower drill bit sizes were used. This was of theoretic interest for future applications as the HEARO procedure is currently based on the use of 1.8 mm drill bits, but providing the HEARO robot with narrower drill bit sizes in the future, extension of candidacy seems achievable.

Although some image data with a slice thickness of up to 0.5 mm were sufficient enough to plan a trajectory, bad imaging resolution was the most frequent reason for a failure in planning, followed by software problems, failure in 3D ear reconstruction, and corrupted image data. A slice thickness of up to 1.3 mm might be enough for CDL planning in some cases (24, 25), but based on the current data and our experience, sufficient visualization of the facial nerve or the chorda tympani is almost impossible. Even in imaging with a slice thickness of 0.3–0.5 mm, the chorda tympani can be only visualized if the angle of the X-ray is in favor of the nerve's location and reconstruction allows for sufficient presentation on the image. None of the scans thicker than 0.5 mm could be used for planning a safe trajectory in the current study.

As a consequence, preoperative assessment should include good image resolution with a slice thickness of maximum 0.3 mm, preferably 0.1–0.2 mm. Otherwise, a high risk of planning failure remains. Considering the exposure to radiation, repetition of scans should be strictly avoided. A standard CT examination protocol for the temporal bone applies an effective dose of ~0.6 millisievert (mSv). By reducing the tube current (milliampere, mA), an effective dose of 0.3–0.5 mSv can be achieved without the loss of diagnostic information (26, 27), which is the case for one scan in the robotic CI set-up. Up until now, two to three scans are necessary during robotic CI surgery; one for assessment of the head with fiducials drilled to the bone before the drilling of the HEARO procedure starts and another scan is performed for safety reasons before the facial recess is entered by the drill bit. With increased experience in robotics in the future, fewer scans might be necessary and could reduce exposure to radiation.

Software crashes counted for some planning failures but with regularly offered software updates, this should not pose a problem in the future. A few patients had malformations of the middle ear such as missing ossicles, resulting in failure of 3D ear reconstruction because segmentation of software-requested landmarks was not possible. One patient was already implanted with a CI contralateral to the measured ear and therefore some steps of the planning course could not be carried out.

One of the advantages expected from robotic ear surgery is planning a safe approach to middle and inner ear structures in case of malformations, which could be a great challenge if the surgery is performed manually. At present, trajectory planning is based on selection of specific landmarks (e.g., selection of the incudostapedial joint). In cases of a malformed middle ear, delineation of those landmarks is currently not possible. Therefore, more flexible measurement procedures should be implemented in order to find a reliable path to the round window.

Although the interrater reliability was good in assessing the trajectory, feasibility of the HEARO procedure with the standard drill bit was less often assessed with observer 1 than with observer two (19 vs. 35 ears). It seems that the measurement procedure differed systematically between both examiners. This further points out that people using this software should be well trained. Case discussions and training lessons on a regular basis should therefore aid consistency of planning results. However, both observers agreed very well on which data was not sufficient enough to plan with the software.

The current results show that the mean CDL of analyzed ears was in the range with previously published data (24, 25). Between well trained examiners, the CDL differed by 1.5 mm on average, which was reported by Canfarotta et al. (28). Here, the absolute difference of the mean CDL between observers was 1.34 mm suggesting strong inter- and intrarater reliability. The ideal insertion angle for different surgical techniques has been demonstrated earlier and would not deviate much from 0° but with given anatomic landmarks, the facial nerve could be harmed (22). Therefore, an optimal trajectory respects vulnerable anatomical structures with the lowest deviation from 0°. In this study, the assessed in-plane angles ranged from 0.1 to 39.5° and calculated out-plane angles ranged between 5.2 and 29°. This is in line with reported optimal out-plane angles varying between −3° and 21° for a posterior tympanotomy approach to the round window and for a given facial recess (22).

A shortcoming of this study is that only four children were included. We primarily collected data of adult patients because at this time, robotic surgery is only accredited in patients older than 18 years due to safety reasons. Concern is raised by the use of radiation before and during the procedure. Children will hopefully benefit from this new technique in the future if acquisition of imaging can be avoided by improving the accuracy of intraoperative facial nerve monitoring. Another limitation is that the time investment for training lessons and study measurements was not assessed and therefore no specific learning curve can be reported.

## Conclusion

Sufficient image resolution, preferably 0.1–0.2 mm slice thickness achieved in low-dose radiation cone beam CT scanners or high-resolution CT, should be performed in the preoperative patient assessment. Otherwise, a high rate of planning failures has to be expected and repetition of scans should not be an option due to unnecessary exposure to radiation. Surgeons should be well and systematically trained in the software planning procedure. With increasing experience in robotic ear surgery, some of the downsides with this new technique - such as exclusion of children - will hopefully be diminished.

## Data Availability Statement

The raw data supporting the conclusions of this article will be made available by the authors, without undue reservation.

## Author Contributions

AA, CA, and DR planned the study. AA and VD performed measurements. DR and W-DB provided help in interpretation of the results. RL performed statistics. AA wrote the main paper. All authors contributed to this work, discussed the results, and commented on the manuscript at all stages.

## Funding

The study was funded by MED-EL GmbH (Innsbruck, Austria). The funding source had no influence on the presented data of this study.

## Conflict of Interest

The authors declare that the research was conducted in the absence of any commercial or financial relationships that could be construed as a potential conflict of interest.

## Publisher's Note

All claims expressed in this article are solely those of the authors and do not necessarily represent those of their affiliated organizations, or those of the publisher, the editors and the reviewers. Any product that may be evaluated in this article, or claim that may be made by its manufacturer, is not guaranteed or endorsed by the publisher.

## Footnotes

^1^https://www.who.int/news-room/fact-sheets/detail/deafness-and-hearing-loss, accessed on May, 21st, 2021.

^2^ https://www.medistat.de/glossar/uebereinstimmung/cohens-kappa-koeffizient

## References

[1] House WF. Cochlear implants. Ann Otol Rhinol Laryngol. (1976) 85:1–93. 10.1177/00034894760850S302 779582

[2] Davies B. A review of robotics in surgery. Proc Inst Mech Eng H. (2000) 214:129–40. 10.1243/0954411001535309 10718057

[3] Marescaux J Leroy J Rubino F Smith M Vix M Simone M. Transcontinental robot-assisted remote telesurgery: feasibility and potential applications. Ann Surg. (2002) 235:487–92. 10.1097/00000658-200204000-00005 11923603 PMC1422462

[4] George EI Brand TC LaPorta A Marescaux J Satava RM. Origins of robotic surgery: from skepticism to standard of care. JSLS. (2018) 22:e2018.00039. 10.4293/JSLS.2018.00039 30524184 PMC6261744

[5] McLeod IK Melder Da PC. Vinci robot-assisted excision of a vallecular cyst: a case report. Ear Nose Throat J Ear Nose Throat J. (2005) 84:170–2. 10.1177/014556130508400315 15871586

[6] Panara K Shahal D Mittal R Eshraghi AA. Robotics for Cochlear Implantation Surgery: Challenges and Opportunities. Otol Neurotol. (2021) 42:e825–35. 10.1097/MAO.0000000000003165 33993143

[7] Warren FM Balachandran R Fitzpatrick JM Labadie RF. Percutaneous cochlear access using bone-mounted, customized drill guides: demonstration of concept in vitro. Otol Neurotol. (2007) 28:325–9. 10.1097/01.mao.0000253287.86737.2e 17414037

[8] Federspil PA Geisthoff UW Henrich D Plinkert PK. Development of the first force-controlled robot for otoneurosurgery. Laryngoscope. (2003) 113:465–71. 10.1097/00005537-200303000-00014 12616198

[9] Danilchenko A Balachandran R Toennies JL Baron S Munske B Fitzpatrick JM. Robotic mastoidectomy. Otol Neurotol. (2011) 32:11–6. 10.1097/MAO.0b013e3181fcee9e 21042227 PMC3064435

[10] Yoo MH Lee HS Yang CJ Lee SH Lim H Lee S. A cadaver study of mastoidectomy using an image-guided human-robot collaborative control system. Laryngoscope Investig Otolaryngol. (2017) 2:208–14. 10.1002/lio2.111 29094065 PMC5655553

[11] Bell B Stieger C Gerber N Arnold A Nauer C Hamacher V. A self-developed and constructed robot for minimally invasive cochlear implantation. Acta Otolaryngol Taylor & Francis. (2012) 132:355–60. 10.3109/00016489.2011.642813 22385333

[12] Torres R Jia H Drouillard M Bensimon J-L Sterkers O Ferrary E. An Optimized Robot-Based Technique for Cochlear Implantation to Reduce Array Insertion Trauma. Otolaryngol Head Neck Surg. (2018) 159:900–7. 10.1177/0194599818792232 30084309

[13] Kontorinis G Lenarz T Stöver T Paasche G. Impact of the insertion speed of cochlear implant electrodes on the insertion forces. Otol Neurotol. (2011) 32:565–70. 10.1097/MAO.0b013e318219f6ac 21478788

[14] Labadie RF Balachandran R Noble JH Blachon GS Mitchell JE Reda FA. Minimally invasive image-guided cochlear implantation surgery: first report of clinical implementation. Laryngoscope. (2014) 124:1915–22. 10.1002/lary.24520 24272427 PMC4453761

[15] Caversaccio M Wimmer W Anso J Mantokoudis G Gerber N Rathgeb C. Robotic middle ear access for cochlear implantation: First in man. PLoS ONE Public Library of Science. (2019) 14:e0220543. 10.1371/journal.pone.0220543 31374092 PMC6677292

[16] Weber S Gavaghan K Wimmer W Williamson T Gerber N Anso J. Instrument flight to the inner ear. Sci Robot. (2017) 2:eaal4916. 10.1126/scirobotics.aal4916 30246168 PMC6150423

[17] Caversaccio M Gavaghan K Wimmer W Williamson T Anso J Mantokoudis G. Robotic cochlear implantation: surgical procedure and first clinical experience. Acta Otolaryngol. (2017) 137:447–54. 10.1080/00016489.2017.1278573 28145157

[18] Anso J Scheidegger O Wimmer W Gavaghan K Gerber N Schneider D. Neuromonitoring during robotic cochlear implantation: initial clinical experience. Ann Biomed Eng. (2018) 46:1568–81. 10.1007/s10439-018-2094-7 30051248

[19] Anso J Dür C Apelt M Venail F Scheidegger O Seidel K. Prospective validation of facial nerve monitoring to prevent nerve damage during robotic drilling. Front Surg. (2019) 6:58. 10.3389/fsurg.2019.00058 31632981 PMC6781655

[20] Gerber N Bell B Gavaghan K Weisstanner C Caversaccio M Weber S. Surgical planning tool for robotically assisted hearing aid implantation. Int J Comput Assist Radiol Surg. (2014) 9:11–20. 10.1007/s11548-013-0908-5 23765213

[21] World Medical Association. World Medical Association Declaration of Helsinki: ethical principles for medical research involving human subjects. JAMA. (2013) 310:2191–4. 10.1001/jama.2013.281053 24141714

[22] Topsakal V Matulic M Assadi MZ Mertens G Rompaey VV Van de Heyning P. Comparison of the surgical techniques and robotic techniques for cochlear implantation in terms of the trajectories toward the inner ear. J Int Adv Otol. (2020) 16:3–7. 10.5152/iao.2020.8113 32209514 PMC7224420

[23] Williamson T Gavaghan K Gerber N Weder S Anschuetz L Wagner F. Population statistics approach for safety assessment in robotic cochlear implantation. Otol Neurotol. (2017) 38:759–64. 10.1097/MAO.0000000000001357 28196000

[24] Spiegel JL Polterauer D Hempel JM Canis M Spiro JE Müller J. Variation of the cochlear anatomy and cochlea duct length: analysis with a new tablet-based software. Eur Arch Otorhinolaryngol. (2021) 1–11. 10.1007/s00405-021-06889-0 34050805 PMC8930796

[25] Cooperman SP Aaron KA Fouad A Tran E Blevins NH Fitzgerald MB. Assessment of inter- and intra-rater reliability of tablet-based software to measure cochlear duct length. Otol Neurotol. (2021) 42:558–65. 10.1097/MAO.0000000000003015 33492059

[26] Lutz J Jäger V Hempel MJ Srivastav S Reiser M Jäger L. Delineation of temporal bone anatomy: feasibility of low-dose 64-row CT in regard to image quality. Eur Radiol. (2007) 17:2638–45. 10.1007/s00330-007-0578-1 17342488

[27] Husstedt HW Prokop M Dietrich B Becker H. Low-dose high-resolution CT of the petrous bone. J Neuroradiol. (2000) 27:87–92. 10970959

[28] Canfarotta MW Dillon MT Buss E Pillsbury HC Brown KD O'Connell BP. Validating a New Tablet-based Tool in the Determination of Cochlear Implant Angular Insertion Depth. Otol Neurotol. (2019) 40:1006–10. 10.1097/MAO.0000000000002296 31290802 PMC6697191

